# Striatal and midbrain connectivity with the hippocampus selectively boosts memory for contextual novelty

**DOI:** 10.1002/hipo.22434

**Published:** 2015-04-09

**Authors:** Alexandros Kafkas, Daniela Montaldi

**Affiliations:** Memory Research Unit, School of Psychological Sciences, University of ManchesterManchester, United Kingdom

**Keywords:** hippocampus, substantia nigra, globus pallidus, novelty, context

## Abstract

The role of contextual expectation in processing familiar and novel stimuli was investigated in a series of experiments combining eye tracking, functional magnetic resonance imaging, and behavioral methods. An experimental paradigm emphasizing either familiarity or novelty detection at retrieval was used. The detection of unexpected familiar and novel stimuli, which were characterized by lower probability, engaged activity in midbrain and striatal structures. Specifically, detecting unexpected novel stimuli, relative to expected novel stimuli, produced greater activity in the substantia nigra/ventral tegmental area (SN/VTA), whereas the detection of unexpected familiar, relative to expected, familiar stimuli, elicited activity in the striatum/globus pallidus (GP). An effective connectivity analysis showed greater functional coupling between these two seed areas (GP and SN/VTA) and the hippocampus, for unexpected than for expected stimuli. Within this network of midbrain/striatal–hippocampal interactions two pathways are apparent; the direct SN–hippocampal pathway sensitive to unexpected novelty and the perirhinal–GP–hippocampal pathway sensitive to unexpected familiarity. In addition, increased eye fixations and pupil dilations also accompanied the detection of unexpected relative to expected familiar and novel stimuli, reflecting autonomic activity triggered by the functioning of these two pathways. Finally, subsequent memory for unexpected, relative to expected, familiar, and novel stimuli was characterized by enhanced recollection, but not familiarity, accuracy. Taken together, these findings suggest that a hippocampal–midbrain network, characterized by two distinct pathways, mediates encoding facilitation and most critically, that this facilitation is driven by contextual novelty, rather than by the absolute novelty of a stimulus. This contextually sensitive neural mechanism appears to elicit increased exploratory behavior, leading subsequently to greater recollection of the unexpected stimulus. © 2015 The Authors Hippocampus Published by Wiley Periodicals, Inc.

## INTRODUCTION

Prompt detection of stimuli whose novelty or familiarity is unexpected in the current environment (i.e., contextual novelty) provides an evolutionary advantage, enabling adaptive behavior and flexible learning of the new information (Kakade and Dayan, 2002). The ability to discriminate potentially salient familiar and novel information, in a context abundant with old and new stimuli, becomes crucial for ensuring effective contextual learning. Efficient discrimination may trigger an orienting response toward the new unexpected information and may therefore enhance memory formation (Tulving and Kroll, 1995). Indeed, the brain's dopaminergic system, including striatal and midbrain structures, has been shown to respond to reward anticipation and to prediction errors as well as having an effect on memory formation (Adcock et al., 2006; Wittmann et al., 2011). It has also been proposed (Lisman and Grace, 2005; O'Carroll et al., 2006; Shohamy and Adcock, 2010) that interaction between the midbrain dopaminergic regions and the hippocampus is crucial for the construction of new memories, especially when novel information is involved (Bunzeck and Duzel, 2006; Bunzeck et al., 2010). Drawing on the measures of eye tracking, subsequent memory, and functional magnetic resonance imaging (fMRI), we further explored this proposal by manipulating the contextual novelty of novel and familiar visual stimuli in three separate experiments.

The response of the dopaminergic midbrain and striatum to unexpected information may be associated with increased exploration of such a stimulus (Düzel et al., 2010), perhaps to establish its *absolute* novelty or familiarity status. Dopamine release, from the midbrain to the evaluation centers in the ventral striatum and from there to the hippocampus, may therefore be manifested behaviorally through an enhanced visual exploration of the presented information, leading to enhanced memory formation for this information. Eye movements have been found to be the markers of the increased visual exploration that leads to enhanced encoding into long-term memory (Kafkas and Montaldi, 2011), whereas pupil responses are argued to reflect resource allocation, cognitive processing (Granholm et al., 1996; Kahneman and Beatty, 1966; for a recent review, see Laeng et al., 2012), and more recently also encoding and retrieval processes (Kafkas and Montaldi, [Bibr b19],[Bibr b20]; Papesh et al., 2012). The current research explored eye movement and pupil response patterns as well as striatal/midbrain and medial temporal lobe (MTL) blood oxygenation level-dependent (BOLD) responses while unexpected novel and familiar stimuli were presented. Critically, in contrast with the previous studies that have focused on the encoding of rewarding information, the current experiments focused on retrieval when familiar and novel, expected and unexpected stimuli were (re-)encoded. The aim of this design was to further characterize the role of the dopaminergic system in memory at retrieval, for both familiar and novel stimuli, when these are either expected or unexpected, as determined by the experimental context. Retrieval provides a setting in which to vary the probability of old and new stimuli, so as to explore how memory is updated depending on both the novelty and the familiarity status of a stimulus, and the interaction of that status with contextual expectation. This investigation promises to provide a more comprehensive, but so far, lacking (Scimeca and Badre, 2012), understanding of the role of the dopaminergic system in memory.

Specifically, the purpose of this research was to investigate the degree to which the detection of contextual novelty (for both novel and familiar stimuli) would trigger an increased response in the striatal and midbrain dopaminergic system and whether this would, in turn, modulate the eye behavior accompanying visual processing, and in so doing, enhance further encoding of contextual information. We hypothesized that if, indeed, striatal and midbrain activity accompanies the processing of contextual novelty this would drive exploratory behavior, reflected in eye movements and pupil responses. Furthermore, we hypothesized that functional connectivity of the striatal/midbrain structures with the MTL, in response to the processing of unexpected (compared to expected) information, would explain the enhanced learning. These hypotheses were explored in two separate, but matched, experiments drawing on fMRI BOLD responses and eye measures (pupil response and fixation patterns). Finally, subsequent memory for unexpected familiar and novel stimuli was explored in a third experiment using a surprise follow-up memory test.

## MATERIALS AND METHODS

### Participants

In the fMRI study 17 (10 females) right-handed volunteers with a mean age of 22.6 years (SD = 4.2) gave informed consent and participated. Remuneration was given at a rate of £20 per session. Another 37 (27 females) undergraduate psychology students, with a mean age of 20.3 years (SD = 2.0) took part in the eye-tracking experiment in return for course credits. In both experiments, volunteers with normal or corrected (with contact lenses) to normal vision were recruited. No history of psychiatric or neurological disorder and no systematic use of psychotropic medicines or drugs were reported by participants in either experiment. Finally, 27 participants (24 females) with a mean age of 20 years (SD = 1.97) participated (receiving £5) in Experiment 3. The experiments were approved by the Research Ethics Committee of the University of Manchester (eye tracking and behavioral) and the National Research Ethics Service (fMRI study).

### Stimulus Materials

The pictures of manmade and natural objects, normalized to a mean gray level, were used in all three experiments. In the fMRI study, 318 of these stimuli were used (18 for practice), whereas the eye**-**tracking experiment used 220 stimuli (20 stimuli for practice), each subtending a visual angle of 18.69° horizontally and 14.03° vertically at presentation. In the behavioral subsequent memory study (Experiment 3), 280 stimuli (220 from the eye**-**tracking experiment plus 60 new foils) were presented. Owing to the sensitivity of pupillary responses to light intensity (Cheng et al., 2006), as well as to chromatic changes (Tsujimura et al., 2006), stimulus properties such as ambient light, brightness, contrast, and the color of the presented visual items were controlled (for further details regarding the development and standardization of these stimuli, see Kafkas and Montaldi, 2011).

### Experimental Design and Task

**Figure 1:**
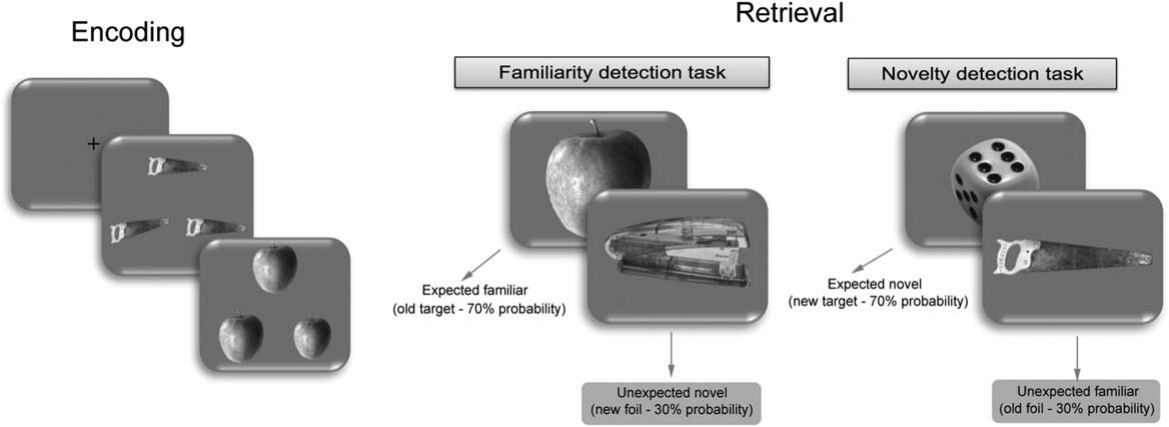
Experimental design of the fMRI (Experiment 1) and eye-tracking (Experiment 2) studies.

In the fMRI and eye-tracking experiments, participants completed two phases: an encoding phase and a retrieval phase, with the fMRI and eye-tracking data recorded during the retrieval phase. At encoding, a series of visual stimuli were encoded using a perceptual-matching-to-sample task with each stimulus appearing for 4 s. In each trial, participants were presented with triplet images of the same object and were asked to decide which of the two bottom images was identical to the target image presented on top (Fig. 1; for this task, see Montaldi et al., 2006; Kafkas and Montaldi, [Bibr b20],[Bibr b21]). After a short filled interval (∼10 min), participants were presented with two recognition conditions, a novelty task (NT) emphasizing the detection of novel stimuli, and a familiarity task (FT) emphasizing the detection of familiar stimuli. Critically, 70% of the items presented in the two tasks were the “target” stimuli (i.e., novel in the NT condition and familiar in the FT condition), whereas 30% of the items in each task were foils (familiar stimuli in the NT condition and novel stimuli in the FT condition; Fig.1). These foil stimuli, which were presented with considerably lower probability, are classified here as unexpected items, compared to the target familiar and novel stimuli, which are classified as expected. For each target stimulus, participants provided a rating of how strongly familiar (FT) or novel (NT) they felt they were. In response to the unexpected stimuli (foils), on the other hand, participants were only required to report them as familiar or novel. The comparison of the BOLD response (Experiment 1) and the eye-tracking measures (Experiment 2) produced by unexpected versus expected novel and familiar stimuli is therefore the main focus of this study.

All stimuli were presented for a period of 3 s during which participants responded. In the fMRI experiment, 6 alternating blocks of familiarity (FT) and novelty (NT) conditions were presented, whereas in the eye-tracking experiment the two tasks were presented sequentially in an order that was counterbalanced across participants. In the fMRI study, 60 null events (providing an implicit baseline) were intermixed with the 300 visual stimuli, pseudorandomly allocated across the six blocks with the only restriction being that null events were never presented consecutively. These null events involve a fixation cross presented centrally and constitute therefore slightly extended presentations of the interstimulus fixation cross. This ensures that the null events are implicitly processed as they are not distinguished from standard interstimulus presentations by participants. A debriefing procedure after the end of the fMRI session indicated that no participant perceived the null events as unexpected in any way.

The design of the behavioral follow-up study (Experiment 3) was identical to the eye-tracking experiment with the only difference being the addition of a surprise retrieval task at the end in which memory for all the stimuli presented in the two main recognition conditions (FT and NT) was tested. Due to the addition of the surprise recognition procedure, the number of stimuli presented in the two main recognition conditions (FT, NT) was reduced maintaining, however, the same probability (30%) for new and old foils in FT and NT, respectively. The completion of the familiarity and novelty tasks was followed by a 10-min distracter task, after which participants were presented with the surprise recognition memory test in which 120 stimuli from the FT and NT conditions were presented together with 60 new foils. Participants were instructed to provide familiarity (F) or recollection (R) responses to each stimulus if they believed they had seen it at some point in the experiment, or to report it as new, if they felt they were encountering the stimulus for the first time (remember/know procedure; for the instructions used see Migo et al., 2012).

### fMRI and Eye-Tracking Data Acquisition

MR scanning was carried out on a 3T MRI scanner (Philips, Achieva) using a gradient echo-planar imaging (EPI) sequence to obtain T2* images, employing the BOLD contrast. Forty slices positioned parallel to the AC–PC line and covering the whole brain (matrix size, 96 × 96; voxel size, 2.5 × 2.5 × 3.5 mm) were acquired for each volume (TR = 2.5 s; TE = 35 ms). In total, 726 volumes were recorded per participant across two sessions (363 per session). High-resolution T1 anatomical images (size, 256 × 256; 180 slices; size: 1 mm isotropic) were acquired for each participant before the two functional sessions.

In the eye-tracking study, pupillary responses and eye movements were recorded using an ASL infrared eye-tracking system (Applied Science Laboratories, Bedford, MA; Model Eye-Trac 6000; sampling rate 60 Hz). A nine-point standard calibration procedure was carried out separately for each participant prior to data collection. For each trial, the eye-tracking data were recorded and analyzed from stimulus onset until the point of behavioral response. Blinks and other losses in the raw eye signal were identified by the eye-tracking software and were discarded from further analyses. Pupil recordings departing more than 3 SDs from the mean of each trial were discarded, as such abrupt pupil dilations or constrictions are considered noise in the pupil signal (Granholm et al., 1996; Beatty and Lucero-Wagoner, 2000). Trials containing <60% of valid recordings were excluded (<5% of total). The peak pupil size for each trial was calculated as the average of three pupil recordings preceding and three recordings following the maximum pupil value of the corresponding trial. The resulting peak pupil responses for each trial of the experiment were expressed as the deviation from the baseline pupil size, recorded during a period of 1,000 ms preceding each trial (Beatty and Lucero-Wagoner, 2000), consisting of a gray background (RGB level, 130). Finally, from the raw eye movement signal, a measure of spatial fixation (i.e., the number of fixations) was extracted.

### Data Analyses

#### fMRI preprocessing

The fMRI data were preprocessed using SPM8 (Statistical Parametric Mapping, Wellcome Department of Cognitive Neurology, University College London, London, United Kingdom; http://www.fil.ion.ucl.ac.uk/spm/). The EPI data were realigned to the first image and resliced using a six-parameter rigid body transformation and sinc interpolation in space. Slice-timing correction was also applied to the resliced images, which were then spatially normalized to the Montreal Neurological Institute (MNI) EPI template. Smoothing was performed using an isotropic (FWHM, 6 mm) Gaussian kernel.

A canonical hemodynamic response function (Friston et al., 1998) was used to model the event-related responses for each participant. Correctly recognized expected and unexpected familiar and novel stimuli were modeled as separate conditions, whereas regressors of no interest (e.g., cue screens preceding each block, trials with no response, etc.) were also included in each individual model. The six movement parameters for each of the two sessions of the experiment were also modeled as regressors to capture residual movement-specific variance in the time series.

#### fMRI data analyses

Contrasts of the *t-statistic* for the four conditions of interest (expected novelty, unexpected novelty, expected familiarity, and unexpected familiarity) were produced for each participant in the first-level analysis, and carried forward to a second-level group analysis treating participants as a random (mixed) effect. A two-way ANOVA was conducted in SPM with the stimulus type (familiar or novel) and expectation (expected or unexpected) as the within-subjects factors. Two direct *t* contrasts (unexpected novelty > expected novelty and unexpected familiarity > expected familiarity) were used to further explore the functional data. Activations surviving a cluster-wise *P* < 0.05 significance level (cluster FWE-corrected) are reported unless otherwise stated. MNI coordinates are reported for the activation data.

#### Psychophysiological interaction analyses

Effective connectivity was explored using psychophysiological interaction (PPI) analysis in SPM8 (Friston et al., 1997). PPI models were used to explore the hypothesis that the detection of unexpected familiar or novel stimuli would be associated with increased connectivity between the midbrain/striatal areas and the MTL. A PPI analysis explores the effect of activity in one brain region, on the activity in another region under the influence of a psychological contrast (i.e., unexpected vs. expected events). A significant PPI between two brain regions indicates that the slope of the regression characterizing their activity changes owing to the effect of the psychological context (Friston et al., 1997). Two areas, within the globus pallidus (GP) and the substantia nigra/ventral tegmental area (SN/VTA), showing selective responses to unexpected familiarity and novelty respectively (Results section) were chosen as the two functional seeds. Spheres of 6 mm radius within the GP (centered at *x* = 17, *y* = 1, *z* = −5) and SN/VTA (centered at *x* = 7, *y* = −20, *z* = −8) were created for each participant and the deconvolved BOLD activation data were extracted from these volumes of interests. Two separate PPI analyses were set up for the two seed areas, one for unexpected familiarity (contrast: unexpected familiar > expected familiar stimuli; seed area: GP) and another for unexpected novelty (contrast: unexpected novel > expected novel stimuli; seed area: SN/VTA).

At the first-level analysis, three conditions were entered for each participant: (a) the BOLD signal time series of the seed region (*physiological variable*), (b) the contrast of interest (unexpected > expected familiarity or novelty; *psychological variable*), and (c) the interaction term expressed as the multiplication of the psychological and physiological variables (*PPI*). A general linear model, with these three regressors, was estimated and *t*-contrasts of the interaction term were created for each participant and analyzed at the group level using a random-effects model (for further details, see Friston et al., 1997). The same statistical inference criteria as those applied to the main fMRI analyses were used for identifying significant activations. The MTL regions are reported as significant when the activation survived a FWE small-volume correction for the volume of the MTL or when eight or more contiguous voxels are active at *P* < 0.001. This level was established as being equivalent to a corrected probability level of *P* < 0.05 for the volume of the MTL using a Monte Carlo simulation (implemented in the AlphaSim tool in AFNI).

#### Eye-tracking data analysis

The fixation and pupil data were analyzed using a 2 × 2 repeated measures ANOVA with stimulus type (familiar, novel) and expectation (expected, unexpected) as the within-subject factors. Direct pairwise comparisons are also reported, where relevant, adopting a significance level of *P <* 0.05 (Bonferroni corrected).

#### Follow-up surprise recognition memory experiment analysis

Data from the follow-up recognition memory experiment (Experiment 3) were analyzed to determine whether the unexpected novel and familiar stimuli were later remembered better than the expected novel and familiar stimuli. Using signal detection, *d*′ scores were calculated for familiarity and recollection responses provided at later test for the originally expected and unexpected novel and familiar stimuli. Two ANOVAs were run separately for the novel and familiar stimuli, with response in the follow-up task (familiar or recollected) and expectation status in the original task (expected or unexpected) as the within-subjects factors. Paired *t*-tests were also employed to further explore the significant interactions using a significance level of *P* < 0.05 (Bonferroni corrected).

## RESULTS

### Experiment 1: fMRI Results

Behavioral data acquired during the fMRI study showed that the participants were able to accurately categorize novel and familiar stimuli in both tasks with well above chance accuracy (0.76 ± 0.08 and 0.77 ± 0.06). The analysis of the response times (RTs) for novel and familiar expected and unexpected stimuli showed a significant main effect of expectation (*F*_1,16_ = 5.50, *P* = 0.03). Expected stimuli (familiar_expected_: *M* = 1,749 ms, SD = 236 ms, and novel_expected_: *M* = 1,839, SD = 198 ms) had longer RTs than the unexpected ones (familiar_unexpected_: *M* = 1,679 ms, SD = 193 ms, and novel_unexpected_: *M* = 1,714 ms, SD = 220 ms).

**Figure 2:**
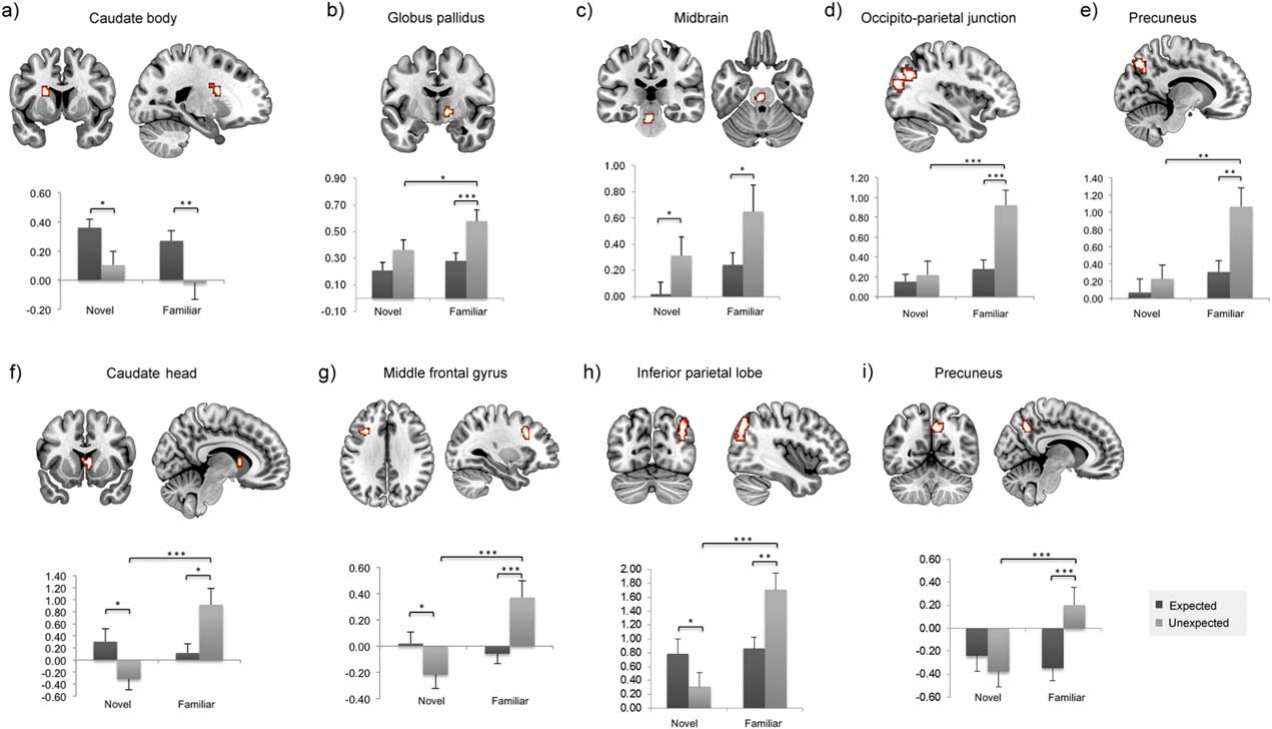
fMRI results from Experiment 1. A main effect of expectation status (expected/unexpected) was observed within (a) the left caudate body *x* = −21, *y* = 6, *z* = 20 (b) the right GP *x* = 15, *y* = −7, *z* = −1, (c) the left midbrain *x* = −3, *y* = −27, *z* = −26, (d) the left occipito-parietal junction *x* = −36, *y* = −70, *z* = 31; BA39 and BA19 and (e) the left precuneus *x* = −11, *y* = −75, *z* = 45; BA7. A significant interaction between expectation status (expected/unexpected) and stimulus type (familiar/novel) was observed in (f) the right caudate head *x* = 5, *y* = 11, *z* = 6, (g) the left middle frontal gyrus *x* = −31, *y* = 16, *z* = 34; BA8/9, (h) the right inferior parietal lobe *x* = 42, *y* = −72, *z* = 48; BA39 and (i) the right precuneus *x* = 12, *y* = -57, *z* = 41; BA7. Parameter estimates are plotted for each region separately. Error bars show one standard error of the mean. Asterisks denote statistically significant effects; *P < 0.05; **P < 0.01; ***P < 0.001. [Color figure can be viewed in the online issue, which is available at wileyonlinelibrary.com.]

The fMRI analyses revealed striatal, midbrain, and neocortical activations for the main effect of expectation as well as for the expectation by stimulus-type interaction (Fig. 2). Four sets of neural response emerged. First, one region within the left caudate nucleus (caudate body, Fig. 2a) responded selectively to expected familiar and novel stimuli compared to unexpected stimuli. A second set included areas within the midbrain/brainstem (Fig. 2c) and the inferior temporal gyrus (BA 37; *x* = −53, *y* = −52, *z* = −19; 37 voxels, *P* < 0.001 uncorrected), which responded to both familiar and novel unexpected stimuli relative to expected ones. A third set of regions, within the right GP (Fig. 2b), the left inferior parietal lobe (extending into the occipito-parietal junction; Fig. 2d), and the left precuneus (Fig. 2e) showed selective involvement in the processing of unexpected familiarity when compared to expected familiarity, but no modulation for novelty. Finally, the interaction effect revealed a set of regions in the right caudate (caudate head, Fig. 2f), the left middle frontal gyrus (Fig. 2g), the right inferior parietal lobe (Fig. 2h), and the right precuneus (Fig. 2i) that respond to unexpected familiarity (relative to expected familiarity) and to expected novelty relative to unexpected novelty. Critically, in these latter regions unexpected familiarity responses were significantly higher than unexpected novelty responses, whereas there were no significant differences between expected familiar and expected novel stimuli. This pattern of results suggests that the regions isolated in the interaction analysis have a predominant role in responding to unexpected familiarity.

**Figure 3:**
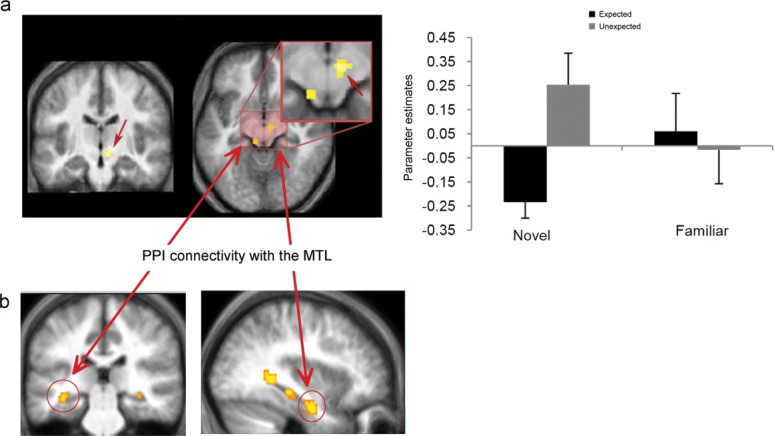
(a) Activity and parameter estimates within SN/VTA (*x* = 7, *y* = −20, *z* = −8) for unexpected novelty and (b) PPI connectivity with the hippocampus (*x* = −36, *y* = −32, *z* = −12 and *x* = −38, *y* = −12, *z* = −26). [Color figure can be viewed in the online issue, which is available at wileyonlinelibrary.com.]

**Figure 4:**
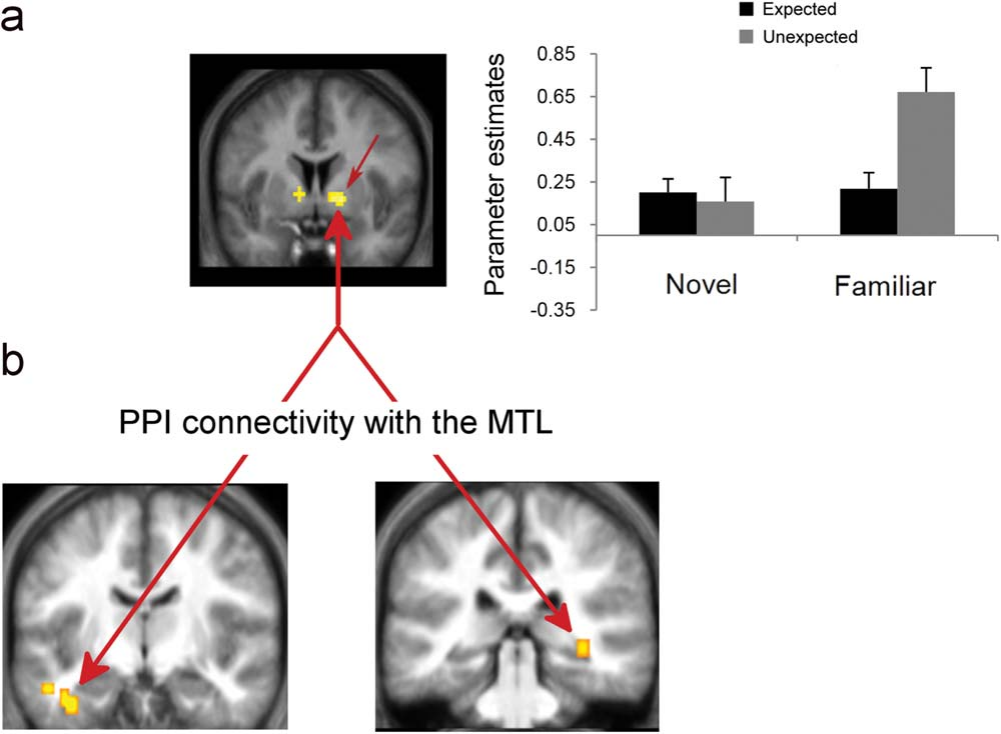
(a) Activity and parameter estimates within the bilateral ventral striatum/GP (*x* = 17, *y* = 1, *z* = −5 and *x* = −13, *y* = −2, *z* = −1) for unexpected familiarity and (b) PPI connectivity with the perirhinal cortex (x = -31, y = -5, z = -33) and the hippocampus (*x* = 35, *y* = −35, *z* = −5). Parameter estimates are plotted for the right GP. [Color figure can be viewed in the online issue, which is available at wileyonlinelibrary.com.]

To further constrain these effects, direct contrasts between unexpected and expected familiar and novel stimuli were run separately. The unexpected novelty versus expected novelty revealed significant activity in the right SN (*x* = 7, *y* = −20, *z* = −8, *P* < 0.001 uncorrected, 21 voxels, *Z* = 4.17; Fig. 3a). The same contrast (unexpected vs. expected) for familiar stimuli revealed significant activity in the bilateral lentiform nucleus (including GP and putamen; Fig. 4a; Right: *x* = 17, *y* = 1, *z* = −5, cluster FWE-corrected; Left: *x* = −13, *y* = −2, *z* = −1, *P* < 0.001 uncorrected, 13 voxels) extending into the thalamus (*x* = 10, *y* = −10, *z* = −1) and the caudate nucleus (*x* = 7, *y* = 6, *z* = −5). In the whole-brain analysis, consistent with the ANOVA effects, the bilateral angular gyri (BA 39; left: *x* = −38, *y* = −72, *z* = 38; right: *x* = 42, *y* = −70, *z* = 41), the bilateral middle occipital gyri (BA 19; left: *x* = −36, *y* = −67, *z* = 24; right: *x* = 37, *y* = −77, *z* = 27), and the bilateral precuneus (BA 7; left: *x* = −6, *y* = 72, *z* = 41; right: *x* = 12, *y* = −57, *z* = 34) also showed significant activity (*P* < 0.05 cluster FWE-corrected) for the unexpected versus expected familiar stimuli.

### PPI: Effective Connectivity Analysis

A PPI analysis with the right SN cluster as the seed area (centered at *x* = 7, *y* = −20, *z* = −8), for unexpected versus expected novelty, showed significantly increased connectivity with the left hippocampus (*x* = −36, *y* = −32, *z* = −12; *P* < 0.001, 8 voxels and *x* = −38, *y* = −12, *z* = −26; *P* < 0.001, 16 voxels; Fig. 3b). The connectivity analysis of the right ventral striatum/GP (centered at *x* = 17, *y* = 1, *z* = −5) showed increased connectivity with the right posterior hippocampus (*x* = 35, *y* = −35, *z* = −5; *P* < 0.001, 8 voxels) and the left PRC (*x* = −31, *y* = −5, *z* = −33; *P* < 0.001, 40 voxels; Fig. 4b) for unexpected versus expected familiarity. Both PPI analyses revealed increased connectivity between the two seed areas and the MTL structures, but no other cortical or subcortical effect was found.

### Experiment 2: Eye-Tracking Results

To further investigate the behavior that accompanies the processing of unexpected familiar and novel stimuli, we analyzed the pupil responses and fixation patterns produced while participants processed these stimuli. In the eye-tracking experiment (Experiment 2), familiar and novel stimuli in the two tasks were categorized with a mean accuracy of 0.81 (SD = 0.07) and 0.82 (SD = 0.10). Consistent with the fMRI experiment, the detection of expected stimuli was accompanied by longer RTs than unexpected ones as indicated by the main effect of expectation (*F*_1,36_ = 28.87, *P* < 0.001; familiar_expected_: *M* = 1,562 ms, SD = 229 ms; familiar_unexpected_: *M* = 1,434 ms, SD = 315 ms; novel_expected_: *M* = 1,591 ms, SD = 261 ms; novel_unexpected_: *M* = 1,394 ms, SD = 228 ms).

**Figure 5:**
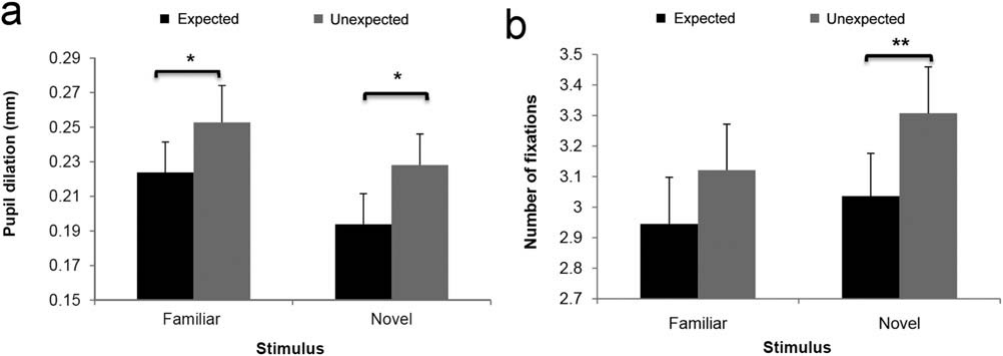
Pupil dilation (a) and fixation patterns (b) for expected and unexpected familiar and novel stimuli in Experiment 2. Error bars show one standard error of the mean; *P < 0.05; **P < 0.01.

The stimulus by expectation ANOVA of the peak pupil dilation produced a significant main effect of stimulus (*F*_1,36_ = 4.99, *P* = 0.03), showing that the detection of familiar stimuli produced more elevated pupil dilations than did the detection of novel stimuli. The main effect of expectation was not significant (*F* < 1); however, a significant interaction between stimulus and expectation (*F*_1,36_ = 11.16, *P* = 0.002) showed that unexpected familiar stimuli produced larger pupil dilations than expected familiar stimuli (*t*_36_ = 2.21, *P* = 0.03). Similarly, unexpected novel produced larger pupil dilations than expected novel stimuli (*t*_36_ = −2.56, *P* = 0.015; Fig. 5a). The analysis of the number of fixations did not yield any significant main effect (stimulus: *F*_1,36_ = 1.12, *P* = 0.30; expectation: *F* < 1), but produced a significant interaction between the two factors (*F*_1,36_ = 6.67, *P* = 0.014; Fig. 5b), revealing that unexpected novel stimuli were accompanied by more fixations than expected novel stimuli (*t*_36_ = 2.80, *P* = 0.008). The same pattern was also found for unexpected versus expected familiar stimuli, but this difference did not reach significance (*t*_36_ = 1.27, *P* = 0.21).

### Experiment 3: Subsequent Memory for Unexpected Stimuli

Finally, we investigated the extent to which the patterns of midbrain/striatal–MTL connectivity (from Experiment 1) and the eye-tracking patterns that accompanied unexpected stimuli (from Experiment 2) are associated with any memory advantages for unexpected stimuli versus expected ones. In a separate experiment (Experiment 3), participants completed the same experimental procedures as in the previous experiments. Critically, their memory for the originally expected and unexpected familiar and novel stimuli was tested in a surprise recognition memory task at the end of the session (Materials and Methods section). In this task, participants recognized old and new stimuli (from across the original FT and NT conditions) with a mean accuracy of 0.88 (SD = 0.05) and a false alarm (FA) rate of 0.22 (SD = 0.15), giving an overall memory performance (hit rate − FA rate) of 0.66 (SD = 0.19), which was significantly above chance (*t*_26_ = 18.31, *P* < 0.001).

Familiarity *d*′ scores and recollection *d*′ scores for originally expected and unexpected stimuli were entered into a 2 × 2 ANOVA separately for originally familiar and novel stimuli. Both ANOVAs showed a significant expectation by response interaction for novel (*F*_1,26_ = 21.32, *P* < 0.001) and familiar stimuli (*F*_1,26_ = 12.32, *P* = 0.002). As summarized in Table1, processing unexpected familiar stimuli produced subsequently higher levels of recollection accuracy than processing expected familiar stimuli (*t*_26_ = −4.15, *P* < 0.001), whereas there was no difference in the familiarity (*d*′) accuracy for unexpected versus expected familiar stimuli (*t*_26_ < 1). Similarly, processing unexpected novel stimuli produced higher levels of recollection accuracy than expected novel stimuli (*t*_26_ = −4.03, *P* < 0.001), whereas familiarity *d*′ scores were higher for expected than unexpected novel stimuli (*t*_26_ = 3.16, *P* = 0.004).

**Table 1:** Subsequent Familiarity and Recollection Performance (d′) for Unexpected and Expected Novel and Familiar Stimuli

	Response	Unexpected	Expected
Novel stimuli	Recollection	0.53 (0.08)***	0.11 (0.10)
	Familiarity	1.17 (0.08)	1.30 (0.08)**
Familiar stimuli	Recollection	0.72 (0.11)***	0.29 (0.16)
	Familiarity	1.14 (0.12)	1.11 (0.13)

*Note*: The categorization of stimuli as novel and familiar refers to their status in the main recognition task. Familiarity and recollection responses are obtained from the surprise subsequent memory test. Numbers in the parentheses are standard errors of the mean.

*** *P*<0.001.

** *P*<0.01.

## DISCUSSION

The findings from this research expand our understanding of the nature of the behavioral conditions that might trigger increased connectivity between striatal/midbrain and MTL regions during the detection of novelty and the effect of this on later memory. In addition, the research illustrates the role of visual exploration (eye movements) in the facilitation of encoding during the detection of contextual novelty. The main findings can be summarized as follows. First, we confirmed that striatal and midbrain structures (along with some cortical areas, Fig. 2) responded to *contextual* novelty for both familiar and novel stimuli at retrieval. Critically, the detection of unexpected novel versus expected novel stimuli elicited increased activity in the SN/VTA, whereas the detection of unexpected familiar versus expected familiar stimuli was associated with the activity in the striatum (predominantly within the GP and putamen). In addition, these regions displayed increased effective connectivity during the detection of unexpected versus expected stimuli, with important memory formation regions within the MTL; the hippocampus (connecting with both SN/VTA and GP) and the perirhinal cortex (connecting with GP). Finally, a small set of cortical regions (involving frontal and parietal areas) was found to be sensitive to unexpected versus expected familiarity, but no such cortical effect was found for novelty. In Experiment 2, we showed that the detection of unexpected stimuli (familiar or novel) was associated with greater pupillary dilation and a greater number of fixations, denoting enhanced visual exploration and processing of the unexpected information. Both the fMRI connectivity and the eye-tracking findings suggest that enhanced learning during the detection of unexpected familiar and novel stimuli might be taking place. This hypothesis was confirmed in the final experiment (Experiment 3), which revealed that both unexpected familiar and unexpected novel stimuli were later remembered with higher recollection accuracy than expected stimuli.

### Connectivity Between Midbrain/Striatum and the Hippocampus is Modulated by Contextual Novelty

According to an influential model proposed by Lisman and Grace (2005), a functional loop between the hippocampus and the SN/VTA underlies long-term memory encoding of new information. According to this view, a novelty signal generated in the hippocampus travels via subcortical connections to the midbrain where it stimulates the VTA neurons. The dopaminergic cells in SN/VTA release dopamine, which converges in the hippocampus resulting in the enhancement of long-term potentiation and leads to the construction of new memories (for an extension of this model, see Lisman et al., 2011). Dopaminergic neurons within the SN/VTA and the ventral/dorsal striatal structures have been linked to positive reward prediction errors (D'Ardenne et al., 2008), reward learning (Schultz, 2013), the processing of unexpected information (Joshua et al., 2009), as well as to the facilitation of the learning of new information (Packard and Knowlton, 2002; Lisman and Grace, 2005; Lisman et al., 2011). Furthermore, SN/VTA has been found to respond to novel stimuli independent of their reward value (Schott et al., 2004), whereas coactivation with the hippocampus has been reported as facilitating long-term memory formation for rewarding or highly motivational stimuli (Wittmann et al., [Bibr b54],[Bibr b55]; Adcock et al., 2006; Bunzeck and Duzel, 2006; Shohamy and Wagner, 2008).

The findings presented here further extend these observations by showing that the activation of SN/VTA and the striatum is not driven at all by the absolute novelty of a stimulus, but by the contextual novelty of both novel and familiar stimuli. Contextual expectation or prediction of novel stimuli has been reported before as modulating the amplitude and the topography of the P3a novelty ERP component (Cycowicz and Friedman, 2007) and as playing a pivotal role in determining the magnitude of novelty-related activity in the MTL (Dudukovic and Wagner, 2007; Bunzeck et al., 2010). However, this is the first time that such a mechanism is revealed in the SN/VTA. In addition, this study illustrates that contextual expectation modulates the *connectivity* between the midbrain or the striatum, and the hippocampus. Specifically, the *effective* connectivity analysis identified a close coupling between midbrain/striatum and MTL structures when unexpected information is detected. *Intrinsic connectivity* between the SN/VTA and the hippocampus has recently been found in a resting-state fMRI study (Kahn and Shohamy, 2013), denoting a functional coupling between the two structures. However, an important question, arising from the current models of midbrain–hippocampal crosstalk (Lisman and Grace, 2005; Lisman et al., 2011), asks under what conditions such a coupling is utilized to boost memory formation for novel or even familiar information.

The results of the current PPI connectivity analysis suggest that the close coupling between the dopaminergic midbrain and the hippocampus is modulated by stimulus expectancy and is especially responsive to *contextual* novelty. This means that the suggested hippocampal–SN/VTA loop (Lisman and Grace, 2005) becomes strengthened under conditions when unexpected information is detected and less so when predictable or expected information is detected (whether novel or familiar). Indeed, expected novel stimuli did not yield midbrain activity when contrasted with expected familiar stimuli. Furthermore, a contrast between unexpected familiar stimuli and expected novel stimuli resulted in significant activity in the SN/VTA (*x* = 2, *y* = −27, *z* = −22, cluster FWE-corrected). This indicates that activity in the SN/VTA as a response to the detection of *contextual* novelty even for familiar stimuli exceeds any SN/VTA response to the absolute novelty of a stimulus alone.

It should be noted here that while a few previous studies (Schott et al., 2004; Adcock et al., 2006; Wittmann et al., 2007; Shohamy and Wagner, 2008) have reported concurrent activation of the hippocampus and the midbrain/striatal system for novel stimuli, especially when these are contrasted with familiar stimuli, coactivation indicates activity synchronization between regions, it does not denote functional coupling (or indeed effective connectivity) between the two structures. In contrast, a PPI (as illustrated here) indicates that the slope of the BOLD response observed in two regions (i.e., SN/VTA and the hippocampus) is modulated by the psychological context (in this case, unexpected vs. expected novelty or familiarity; Materials and Methods section). Although, not precisely identified by the fMRI data, it can be assumed that the character of this midbrain/striatum–hippocampal connectivity is dopaminergic (Eckart and Bunzeck, 2013). Therefore, the present findings provide especially strong support for the proposed functional memory circuit linking the dopaminergic midbrain and the hippocampus (Lisman and Grace, 2005; O'Carroll et al., 2006; Shohamy and Adcock, 2010). Furthermore, this circuit appears to exclusively engage striatal/midbrain and MTL regions without the effect of any other intervening brain structures as revealed by the lack of any significant PPI connectivity of SN/VTA and GP with any other cortical or subcortical region. This makes it highly unlikely that the increased connectivity between the midbrain/striatum and the hippocampus arises from the common influence of a third region.

It remains to be established whether there are any functional differences in the contribution made by the ventral striatum (GP) and the SN/VTA to the detection of unexpected incoming information and, critically, whether the nature of their connectivity with the hippocampus is modulated by stimulus type (i.e., nominally novel or familiar). As noted above, SN/VTA and midbrain activity (see also Fig. 2b) appears to respond to both unexpected novel and unexpected familiar stimuli. In contrast, GP activity selectively signals the detection of unexpected familiar stimuli although this activity most likely also originates from the dopaminergic midbrain. Indeed, the ventral pallidum receives major afferent inputs from the dopaminergic midbrain neurons (Haber et al., 1995) and it is very likely that the activity of the dopaminergic neurons in the midbrain results in the modulation of BOLD activity in regions to which these neurons project (Yacubian et al., 2006), such as the GP. Therefore, the connectivity patterns between these two regions (GP and SN/VTA) and the hippocampus, during the detection of contextually unexpected stimuli, may suggest two distinct routes within the striatal/VTA–hippocampus loop.

More specifically, and based on the connectivity findings from this study, a direct projection between the SN/VTA and the hippocampus appears to be employed during the detection of unexpected novel stimuli. This pathway may be considered a reciprocal one, with the hippocampus signaling the detection of contextual novelty to the VTA and the dopaminergic neurons of the VTA projecting their response back to the hippocampus to trigger encoding. This is consistent with the documented role of the hippocampus in novelty detection (Knight, 1996; Köhler et al., 2005; Stoppel et al., 2009; Kafkas and Montaldi, 2014). In contrast, midbrain activity for unexpected familiar stimuli seems to originate from other key memory-related brain regions such as the perirhinal cortex, which showed increased connectivity with the GP for unexpected familiar stimuli, and this signal might project to the hippocampus through the ventral striatum (GP). The selectivity of the deployment of the GP for the processing of unexpected familiar stimuli may reflect the onset of an evaluation process that computes the significance of the incoming familiar information in terms of the need for further encoding by the hippocampus. Therefore, although the hippocampus is not involved in the detection of familiar stimuli (Yonelinas et al., 2005; Montaldi et al., 2006; Kafkas and Montaldi, 2012), it responds to the novelty of the unexpected familiar information through its connectivity with the ventral striatum. Indeed, neurons in the monkey GP have been reported to respond not only to the learning, but critically to the evaluation of the reward value of an action (Pasquereau et al., 2007) and are responsible for initiating the transmission of this signal to dopaminergic centers like the SN/VTA (Hong and Hikosaka, 2008; see also Tecuapetla et al., 2009).

### Greater Visual Exploration and Learning Facilitation for Unexpected Stimuli

What is the functional significance of a mechanism whereby midbrain/striatum activation and its connectivity with the hippocampus is selectively strengthened for unexpected stimuli and how is it manifested behaviorally? We hypothesized that midbrain and striatal activations may trigger enhanced visual exploration and processing of unexpected stimuli (see also Düzel et al., 2010). It has been shown earlier that less frequent or unanticipated events attract greater pupil dilation even when the cognitive demands of the task are kept minimal (Richer and Beatty, 1987; Reinhard and Lachnit, 2002a). This effect vanishes only when the anticipation of an event plays a less crucial role or when other distinctive features of a stimulus become more salient (Reinhard and Lachnit, 2002b; Reinhard et al., 2007). Indeed, in Experiment 2 we showed that the unexpected familiar and novel stimuli were accompanied by increased pupil responses and that unexpected novel stimuli were characterized additionally by increased numbers of fixations compared to those triggered by expected novel stimuli (Fig. 2). These findings suggest that the contextually novel stimuli, which are accompanied by striatal/midbrain responses and enhanced connectivity with the hippocampus, are also characterized by enhanced visual exploration and processing.

Based on these findings, we hypothesized that the SN/VTA triggered hippocampal activity and the richer visual exploration of the unexpected stimuli would lead to enhanced subsequent memory for those items. Experiment 3 confirmed that unexpected stimuli (whether familiar or novel) were recollected later with higher accuracy than expected familiar and novel stimuli. These findings are consistent with the hypothesis that the role of the enhanced midbrain/striatal–hippocampal connectivity is to facilitate learning for unexpected stimuli (see also Lisman et al., 2011) and that this is supported by an enhanced visual exploration of these stimuli (as indicated in Experiment 2). Importantly, as it might be expected from the enhanced connectivity between the midbrain/striatum and the hippocampus, a key region for encoding subsequently recollected events (Ranganath et al., 2004; Staresina and Davachi, 2008), the memory enhancement for unexpected stimuli was selective to recollection memory with no enhancement of familiarity responses.

It should be noted here that the enhanced pupil response and number of fixations characterizing the unexpected stimuli and the ensuing memory benefit cannot be explained by a RT confound. It might have been argued, for example, that the detection of unexpected information is more effortful and thus slower, leading to greater memory for these stimuli owing to the extra time allocated to processing these stimuli. However, in both experiments, the detection of unexpected familiar and novel stimuli was faster than the detection of expected familiar and novel stimuli. Therefore, neither increased effort nor extra processing time can account for the findings in the experiments reported here. The faster RT found for unexpected stimuli relative to expected ones may relate to the differential emphasis put on familiar and novel stimuli within the two retrieval conditions (FT and NT, respectively), or it may result from the engagement in a strength-rating task in the case of target (expected) but not the unexpected stimuli.

One limitation of the presented studies, however, is that the fMRI and the eye-tracking data are derived from different experiments. Nevertheless, absolutely matched experimental designs were adopted in both studies with the only difference being that the fMRI data were acquired while participants lay in the MRI scanner, whereas the eye-tracking data were recorded while they were seated in a testing room. We therefore do not believe that this difference could undermine the potential relationship between the results and an intriguing possibility is that visual exploration is the direct product of the enhanced midbrain/striatal activation and connectivity with the hippocampus. Indeed, the activity of SN neurons in the monkey has been found to relate to saccadic eye movements especially when these are directed toward rewarded locations (Handel and Glimcher, 2000). More recently, neurons in monkey GP demonstrated synchronized activity with saccadic generation (Shin and Sommer, 2010). Therefore, it can perhaps be concluded that the memory facilitation for unexpected stimuli, driven by the hippocampal–midbrain system (Lisman and Grace, 2005), is mediated by, or at the very least correlates with, the modulation of eye movement behavior illustrated in Experiment 2.

### Unexpected Familiarity: a Role for Parietal Cortex

Apart from the striatal/midbrain activations and connectivity patterns with the hippocampus, the analyses also identified areas within the inferior parietal lobe, the occipito-parietal junction, the precuneus, and the middle frontal gyrus (Fig. 2) that showed a selective response to unexpected versus expected familiar stimuli. The parietal regions, in particular, have been previously found to play a role in identifying unexpected stimuli (O'Connor et al., 2010; Jaeger et al., 2013) or stimulus incongruency within a sequence of events (Krebs et al., 2015). For example, in a recent study, Jaeger et al. (2013) found that distinct areas within the inferior parietal lobe monitor unexpected novelty and unexpected familiarity and proposed that their function is to orient current attention to the unexpected information. Our findings are consistent with this model although we observed only inferior parietal activations in response to unexpected familiarity, not to unexpected novelty. In addition, our connectivity data did not show any interactions between the parietal regions and the midbrain/striatal–MTL circuitry. Further studies are needed to explore any communication between the midbrain/striatal–hippocampal circuit and the inferior parietal regions.

## CONCLUSIONS

Taken together, these studies show that unexpected familiar and novel stimuli selectively activate striatal and midbrain regions and are also associated with increased visual exploration and enhanced subsequent memory. Consistent with the theoretical model proposed by Lisman and Grace (2005), this memory facilitation is accompanied by increased connectivity between striatal/midbrain and the hippocampus, and the present findings show that this effect is selective to *contextual* novelty (for both familiar and novel stimuli) not related to absolute stimulus novelty. The findings also discriminate between two potential pathways within this system; one for the detection and processing of unexpected familiar stimuli and the other for the detection and processing of unexpected novel stimuli. Overall, the findings support the hypothesis that striatal/midbrain activity and its connectivity with the hippocampus and other medial temporal lobe structures drives and supports exploratory behavior, subsequently leading to the increased recollection of contextually novel events.
